# Biocatalytic Synthesis of Diamondoid Diols by the Brown‐Rot Fungus *Wolfiporia cocos*

**DOI:** 10.1002/cbic.202500930

**Published:** 2026-02-01

**Authors:** Valeriia V. Nikitenkova, Aryna M. Sydorenko, Holger Zorn, Tatyana Zhuk

**Affiliations:** ^1^ Institute of Food Chemistry and Food Biotechnology Justus Liebig University Giessen Giessen Germany; ^2^ Faculty of Chemical Technology Igor Sikorsky Kyiv Polytechnic Institute Kyiv Ukraine; ^3^ Fraunhofer Institute for Molecular Biology and Applied Ecology Giessen Germany

**Keywords:** biocatalytic oxidation, brown‐rot fungi, diamantane‐1,9‐diol, growing culture

## Abstract

The CH‐oxidative activity of *Wolfiporia cocos* cultures of different ages was studied utilizing diamondoid derivatives as conformationally rigid models. Adamantane‐1‐ol and 1‐bromoadamantane, traditional precursors for the synthesis of polysubstituted adamantanes, gave qualitatively similar reaction mixtures in which adamantane diols dominated. While the reactions occurred with high preparative yields, the biotransformation does not display clear differences from classic electrophilic or radical adamantane functionalization reactions. In contrast, the fungal oxidation of diamantane‐4‐ol occurred with unexpected selectivity yielding diamantane‐4,9‐diol and diamantane‐1,9‐diol. The latter product is quite uncharacteristic for diamantane chemistry because the most deactivated CH‐position is attacked. To elucidate the active fungal component, additional experiments were performed with supernatant and resting cells harvested at various culture ages. Oxidative activity was detected in resting cells from 4‐ and 6‐day‐old cultures in pure water medium, and from 6‐day‐old cultures in buffer at pH 2, but disappeared at higher pH values.

## Graphical abstract

*Wolfiporia cocos* cultures were tested for CH‐oxidative activity using rigid diamondoid substrates. Adamantane derivatives gave typical diols similar to known chemical oxidations, while diamantane‐4‐ol showed unusual selectivity, forming rare 4,9‐ and 1,9‐diols. Tests with supernatants and resting cells showed that oxidation occurred only in cells from 4–6‐d cultures, active in water or at pH 2, but lost at higher pH.

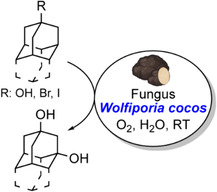

## 1 Introduction

The distinct architecture of diamondoids,characterized by their rigidity and high symmetry, contributes to their exceptional thermal and chemical inertness and lipophilicity [1, 2]. Cage diols, derived from the lower diamondoids, adamantane and diamantane, possess a unique 3D structure that imparts remarkable physicochemical properties. The presence of two hydroxyl groups on the framework expands their synthetic versatility, enabling a wide array of chemical modifications and polymerizations [3]. Such characteristics make cage diols particularly valuable in diverse scientific and industrial applications, ranging from drug discovery and catalyst design to advanced materials science [4, 5, 6, 7, 8, 9, 10, 11]. Among already established examples are acrylic esters of 1‐adamantanol and 1,3‐adamantandiol that are promising as photoresist materials due to their thermal stability and rigidity [12]. Moreover, the *bis*‐hydroxy functionality of adamantane‐1,3‐diol makes it a valuable precursor in the design of photosensitive resins [13], flame retardants [14], and organocatalysts [15, 16]. Its utility in molecular recognition [17, 18] and surfactant chemistry [19] has also been recently demonstrated.

Chemical methods for synthesizing cage diols typically utilize cage hydrocarbons or their mono‐ or disubstituted derivatives. Despite its simple structure, adamantane remains difficult to functionalize as hydroxylation at nonactivated carbon atoms often requires the use of expensive metal catalysts like cobalt, palladium, manganese, etc. along with harsh conditions and toxic reagents (strong oxidants, radicals, or electrophiles). The procedures based on monosubstituted adamantane/diamantane require large excesses of electrophilic reagents leading to several drawbacks, namely increased waste generation, higher costs, and harsh operating environments [20, 21]. Approaches based on disubstituted cage derivatives are based on more expensive precursors and mainly include similar drawbacks. Therefore, conventional laboratory and industrial methods remain inefficient and environmentally unfriendly, and achieving selective C–H functionalization under mild, sustainable conditions continues to be a major challenge [22].

These limitations have redirected research toward biochemical approaches, in which enzyme‐ and whole‐cell‐based systems provide milder, more selective and environmentally sustainable routes for C–H functionalization. A notable early example is cytochrome P‐450cam from *Pseudomonas putida*, which catalyzed the oxidation of adamantane to 1‐adamantanol with high selectivity but limited catalytic efficiency. Whole‐cell systems such as *Streptomyces griseoplanus* [23] and strains of *Absidia* [24, 25] have also been shown to hydroxylate adamantane and 1‐adamantanol, typically yielding mixtures of adamantane‐1,3‐ and 1,4‐diols. However, these approaches often suffer from low selectivity and limited scalability. *Streptomyces* sp. SA8 gave more promising results, efficiently oxidizing 1‐adamantanol to yield predominantly adamantane‐1,3‐diol, with minor amounts of the 1,4‐isomer [26]. Other enzymatic systems have also been reported. Soluble methane monooxygenase from *Methylococcus capsulatus* exhibits a reaction mechanism similar to that of cytochrome P‐450 enzymes. It oxidizes adamantane to 1‐ and 2‐adamantanol in approximately equal proportions [27]. Rabbit liver microsomal cytochrome P‐450_LM2_ shows higher regioselectivity, yielding predominantly 1‐adamantanol with minor formation of 2‐adamantanol and selectively oxidizing adamantanone to 4‐*anti*‐hydroxyadamantanone and 5‐hydroxyadamantanone [28].

Although *Streptomyces* sp. SA8 demonstrated improved selectivity, bacterial systems generally display limited oxidative versatility. This has led to growing interest in basidiomycetous fungi, whose oxidative enzyme toolbox enables a wider range of selective hydroxylation and oxidation reactions under mild conditions. Compared to other microbial systems, fungi exhibit broader substrate tolerance and enhanced oxidative versatility, making them valuable candidates for the biotransformation of cage hydrocarbons and other chemically inert compounds. A recent study demonstrated that *Dichomitus albidofuscus* is capable of oxidizing adamantane with 39% yield in 8 days in the presence of 2‐propanol, suggesting the involvement of metalloenzymes based on positional selectivity and kinetic isotope effect data [29]. In the case of adamantanone, the oxidation proceeds even faster, yielding alcohols and disubstituted derivatives [30]. Under the same conditions, *Pleurotus sapidus*, *Cerrena zonata*, and some other white‐rot fungi also efficiently catalyze the oxidation of adamantanone.

This study is focused on the catalytic C—H bond oxidation by the fungus *Wolfiporia cocos* traditionally utilized in medicinal contexts [31, 32]. *W. cocos* is a brown‐rot fungus, which implies a distinct enzymatic strategy, often characterized by the generation of hydrogen peroxide and the subsequent iron‐mediated Fenton reaction for lignocellulose degradation [33]. This differs from white‐rot fungi that typically employ an array of peroxidases and laccases for lignin digestion [34, 35, 36], highlighting a fundamental divergence in oxidative mechanisms between these fungal groups [37, 38, 39]. Despite these differences, recent omics‐based analyses of *W. cocos* have identified a substantial number of cytochrome P450 enzymes, some of which are likely involved in the synthesis of pychimic acid, namely in the direct enzymatic oxidation of C—H bonds [40]. Apparently, cytochromes are critical for the fungal adaptability and survival, facilitating the detoxification of xenobiotics and the biosynthesis of diverse secondary metabolites, often through regio‐ and stereoselective C—H bond activation [40]. In our recent investigations, *W. cocos* was found to catalyze the oxidation of adamantanone, yielding 5‐hydroxyadamantane‐2‐one and *anti*‐4‐hydroxyadamantane‐2‐one as the major products with 59% and 24% yields, respectively. In addition, minor products including *syn*‐ and *anti*‐adamantane‐1,4‐diols and other dihydroxylated derivatives substituted at secondary positions were detected [41]. This established activity makes *W. cocos* an attractive candidate for further exploration, particularly regarding its potential to transform polyhydroxylated adamantane derivatives.

## 2 Results and Discussion

**SCHEME 1:**
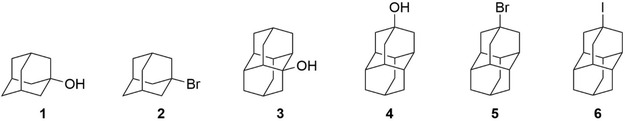
Model compounds as precursors of diamondoid diols: adamantane‐1‐ol (**1**), 1‐bromoadamantane (**2**), diamantane‐1‐ol (**3**), diamantane‐4‐ol (**4**), 4‐bromodiamantane (**5**), and 4‐iododiamantane (**6**).

Previously, it was clearly shown that the presence of electron‐withdrawing substituents in a hydrocarbon cage decreases its reactivity that makes double substitution in the presence of traditional electrophiles rather difficult. We thus focused on the utilization of monosubstituted cage compounds as precursors for the production of adamantane and diamantane diols [42] under biological conditions. Compounds **1**–**6** were tested as precursors towards adamantane and diamantane diols in biocatalytic transformation with *W. cocos* (Scheme 1).

### 2.1 Screening of *W. cocos* Oxidative Activity Toward 1‐Adamantanol

1‐Adamantanol (**1**) has been used as a model compound to examine the most favorable conditions for biotransformations. To evaluate the activity of *W. cocos* cultures toward **1**, cultures of different ages (1–7 days old) were tested. Samples were collected every 24 h over a 6‐day period. In addition, 1‐adamantanol was supplemented at varying concentrations (5, 10, 20, 30 mM).

**FIGURE 1:**
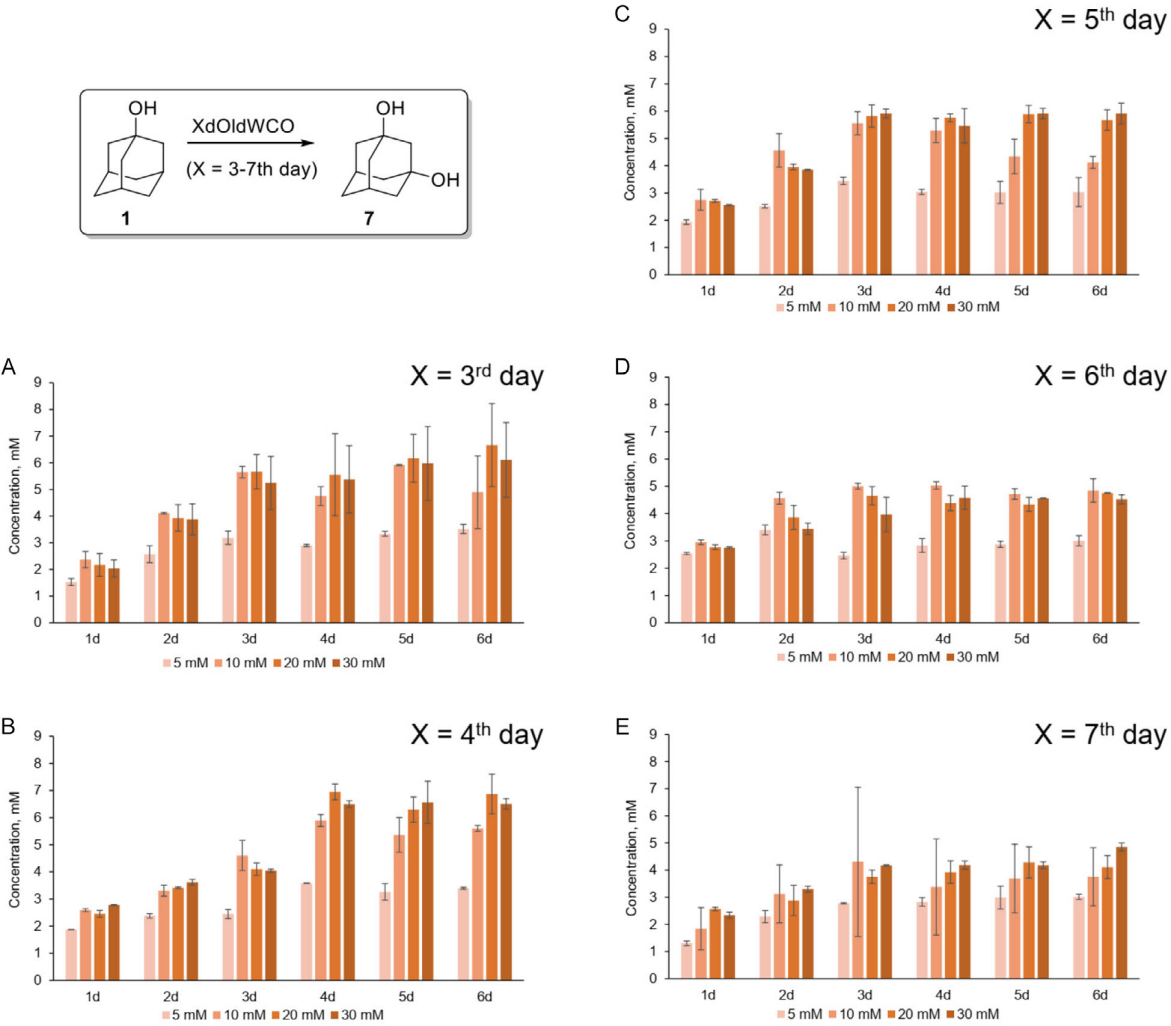
Time course of 1,3‐adamantanediol production in biotransformation reactions with whole‐cell growing cultures of *W. cocos*, using varying concentrations of 1‐adamantanol (5, 10, 20, and 30 mM). Substrate 1 was added to the cultures on the 3^rd^ (A), 4^th^ (B), 5^th^ (C), 6^th^ (D), and 7^th^ (E) day. Error bars represent standard deviations based on triplicate experiments.

The 1‐d‐old cultures showed the highest activity at 5 mM adamantane‐1‐ol (**1**), with the maximum concentration of adamantane‐1,3‐ol (**7**) (0.8 mM) observed on day 4 (Figure S1.1.A). However, culture growth was generally poor, with low mycelial biomass, particularly when supplemented with 10 mM or higher concentration of **1**. In 2‐d‐old cultures, the conversion efficiency improved, reaching its maximum on day 6. At 10 mM of the substrate, the yield of **7** was 5.4 mM. At higher substrate concentrations (20 and 30 mM) the highest yields obtained were 4.8 and 4.3 mM, respectively (Figure S1.1.B). For 3‐d‐old cultures, the maximum product concentration was 6.7 mM at a substrate concentration of 20 and 6.1 mM at 30 mM, both observed on day 6 (Figure 1A). In 4‐d‐old cultures, the highest yield of **7** (6.9 mM) was achieved on day 4 at 20 mM substrate and 6.5 mM at 30 mM. Comparable yields were also detected on day 6 (Figure 1B). A slight decrease in product concentration occurred on day 5, followed by an increase on day 6. This fluctuation likely reflects the limited solubility of 1‐adamantanol in water, leading to its slow dissolution and delayed release into the medium, which temporarily alters substrate availability and, consequently, product formation. At a substrate concentration of 10 mM, the maximum yield of **7** reached 5.9 mM on day 5 in 3‐d‐old cultures and on day 4 in 4‐d‐old cultures, corresponding to ≈59% conversion. In 5‐d‐old cultures, the highest product concentration from 10 mM substrate was 5.5 mM on day 3. For substrate concentrations of 20 and 30 mM, the maximum yields, namely 5.9 mM in both cases, were obtained on day 5. At a 5 mM concentration of **1**, the highest yield of **7** was 3.6 mM on day 4 in 4‐d‐old cultures, corresponding to ≈72% conversion (Figure 1C). In 6‐d‐old cultures, the highest product concentrations were detected from day 3 onward and showed little variation across sampling days (Figure 1D). At a substrate concentration of 10 mM, the product level remained consistently around 5 mM throughout days 3–6. For 20 mM substrate, product concentrations varied only slightly, ranging from 4.3 to 4.8 mM. Similarly, at 30 mM substrate, ≈4.5–4.6 mM of product was measured between days 4 and 6, indicating that further increases in substrate concentration did not significantly enhance product formation. In 7‐d‐old cultures, the product concentration did not exceed 5 mM under any conditions; the highest yield observed was 4.8 mM on day 6 at 30 mM substrate (Figure 1E). This trend suggests that the reduced yields in older cultures may be attributed to a decline in enzyme activity. No products were detected in autoclaved cultures, supporting the conclusion that the C–H oxidation is enzymatically catalyzed.

As a result of above optimizations, the highest yield of **7** was observed in 4‐d‐old cultures (6.9 mM at 20 mM substrate). However, considering the yield of 5.9 mM at 10 mM substrate, it is evident that a 10 mM concentration of the starting compound represents the optimal condition under the tested parameters. Younger cultures showed lower activity, while older ones (6–7 days) produced only about 4.5–5 mM. Product fluctuations were linked to the limited solubility of **1**, and decreased yields in older cultures were likely due to reduced enzyme activity.

It should be noted that *W. cocos* generates highly acidic media during its growth, even lower than that of typical brown‐rot fungi. Previous studies have shown that when grown in malt extract medium, the pH decreased to 2.5 after 7 days [43]. Our measurements indicated that the acidity is even higher reaching 1.78 by day 6 and remained constant after addition of substrate. To enhance substrate solubility, various organic cosolvents, including ethanol, 2‐propanol, and dimethyl sulfoxide, were tested at different concentrations (see Supporting Information). However, even at low solvent concentration, no product formation was observed, indicating that the presence of organic solvents inhibited the biotransformation process.

### 2.2 Screening of *W. cocos* Oxidative Activity toward 1‐Bromoadamantane

Additionally, the transformations of 1‐bromoadamantane (**2**), which undergoes hydrolysis to **1** prior to further oxidation was investigated. This is quite unexpected as the hydrolysis of **2** usually requires harsh reaction conditions, namely refluxing in a DMF/water mixture [44]. Since the screening experiments with **1** revealed that 4‐d‐old cultures exhibited the highest catalytic activity, preparative experiments with **2** were initially conducted using 4‐d‐old cultures. However, only low yields of the target product, 1,3‐adamantanediol (**7**), were obtained with initial concentrations of **2** of 5 and 10 mM. Consequently, additional experiments were performed with 6‐d‐old cultures, which have accumulated more mycelial biomass and presumably developed a more complex enzymatic profile, thereby reducing the toxic effect of the substrate.

**FIGURE 2:**
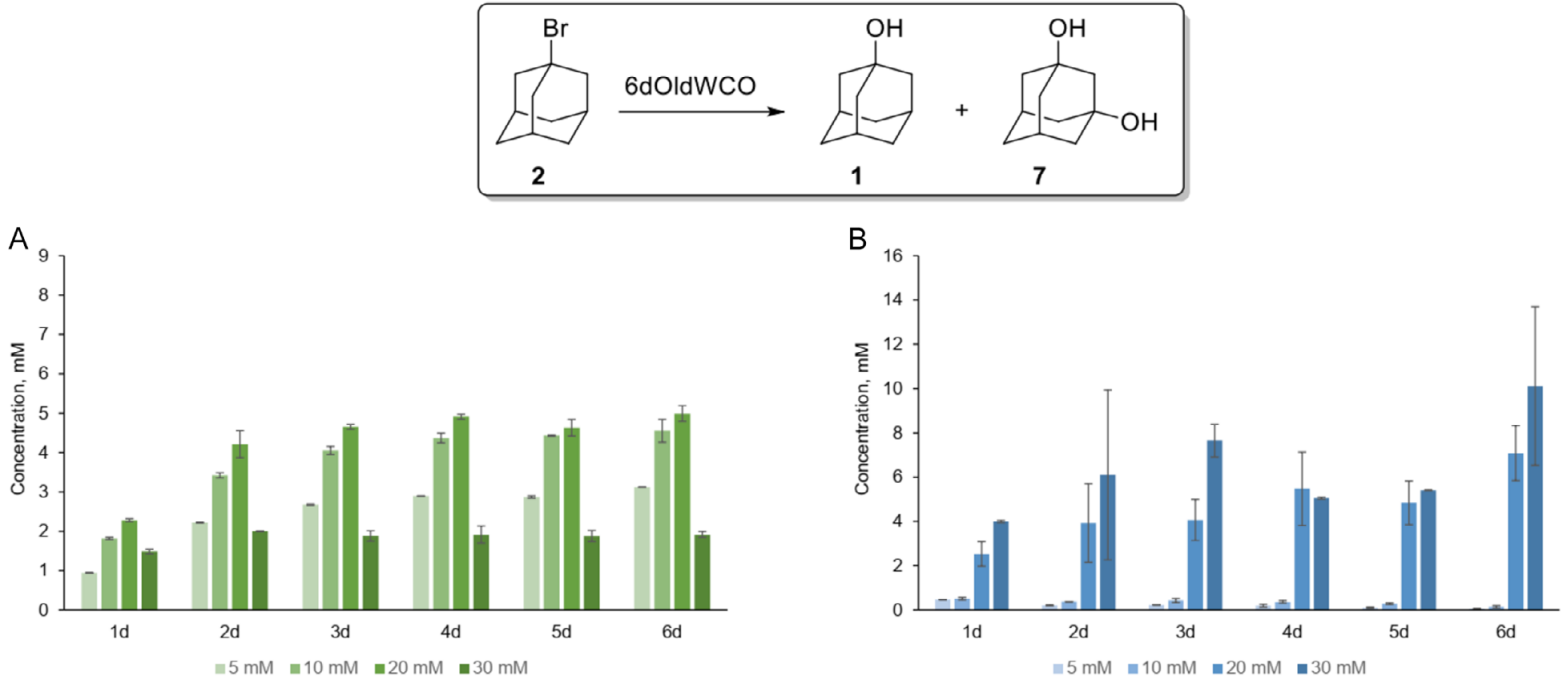
Time course of 1,3‐adamantanediol (A) and 1‐adamantanol (B) production in biotransformation reactions with 6‐d‐old cultures of *W. cocos* with varying concentrations of **2** (5, 10, 20, and 30 mM). Error bars represent standard deviations based on triplicate experiments.

The influence of the substrate concentration on the biotransformation of **2** was examined over an incubation period of 6 days (Figure 2A). At a 5 mM initial substrate concentration, the formation of **7** increased gradually, reaching 3.1 mM by day 6. When the concentration was raised to 10 mM, product **7** levels ranged between 4.0 and 4.5 mM from day 3 onward, with the maximum (4.5 mM) observed on day 6. A similar pattern was observed at 20 mM, where the concentration of **7** remained at 4.6–5.0 mM, reaching a maximum on the day 6. In contrast, at 30 mM substrate, the concentration of **7** did not exceed 2 mM throughout the incubation period, likely due to the substrate's inhibitory or toxic effects at higher concentrations.

Notably, the hydrolysis of **2** also occurred in autoclaved cultures, indicating that it results from the acidic medium rather than enzymatic activity. This is not surprising since solvolysis is accelerated in the presence of protic acids. The formation of **1** *via* nonenzymatic hydrolysis of **2** was also monitored (Figure 2B). Fluctuations in its concentration were attributed to the limited solubility of **2** that causes gradual substrate release and an uneven hydrolysis rate.

### 2.3 Preparative Scale Biotransformation of Model Compounds with *W. cocos*

The transformations of model compounds with *W. cocos* were conducted at 2 mmol scale (300–400 mg) using 200 mL of cultures of different ages depending on the substrate. Based on the screening results, 4‐d‐old submerged cultures were selected for the oxidation of **1** as they exhibited the highest activity under these conditions. In contrast, 6‐d‐old cultures were used for the biotransformation of **2**, since higher product yields were obtained with more mature submerged culture. Our previous study on the oxidation of diamantane ketones showed that more mature *W. cocos* cultures achieved higher substrate conversion, supporting the choice of 6‐day‐old cultures for these transformations [41].

**SCHEME 2:**
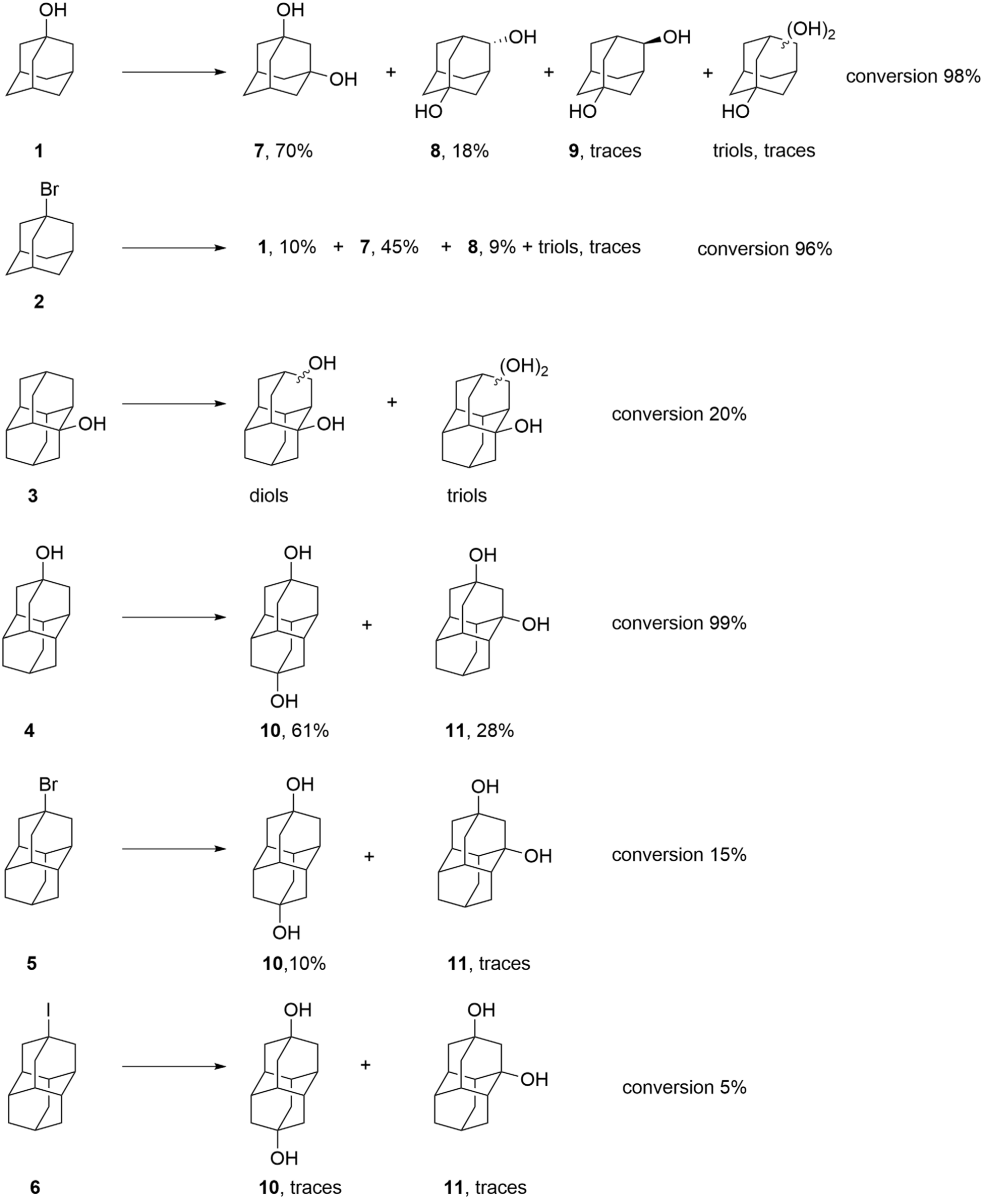
Biotransformations of monosubstituted adamantanes and diamantanes with *W. cocos*. Yields are preparative, traces have been detected by GC–MS.

Both, with **1** and **2**, the conversions were high and **7** was isolated as main product with yields of 70% and 45% correspondingly (Scheme 2). The alcohol diamantane‐1‐ol (**3**) displayed low reactivity as only ca. 20% conversion was observed to give a complex mixture of diamantane diols and triols. Presumably, the presence of an electron‐withdrawing group in the belt position of the diamantane cage reduces the reactivity of the *tert‐*CH bonds of **3**. The diamantane core may also contribute to reduced reactivity by limiting access to enzyme sites due to the increased size of the molecule.

The reactivity of the apical diamondoid alcohol **4** is instructive as the presence of electron‐withdrawing group deactivates the neighboring CH positions, but not the distant apical one. In agreement, the major product of the oxidation of **4** is diamantane‐4,9‐diol (**10**) where the most distant CH group is attacked. However, the formation of substantial amounts of diamantane‐1,9‐diol (**11**) was unexpected as it results from the oxidation of the most deactivated *tert‐*CH position. No traces of the next expected diamantane‐1,4‐diol [45] were detected based on the GC–MS data. We tested the electrophilic functionalization of **4** with 100%‐nitric acid and found **10** as the only product; even trace amounts of **11** were not detected. These results prompted us to study the active components in the fungal cultures that are responsible for such an unusual C–H functionalization mode. The oxidation of 4‐bromodiamantane (**5**) proceeded with a low conversion of approximately 15%. In this case, diamantane‐4,9‐diol (**10**) was isolated in 10% yield, while only trace amounts of diamantane‐1,9‐diol (**11**) were detected. Furthermore, 4‐iododiamantane (**6**) showed even lower reactivity, with a conversion rate of about 5%, and only trace amounts of the diols **10** and **11** were observed. The low reactivity of 4‐halodiamantanes **5** and **6** is consistent with the fact that electron‐withdrawing substituents reduce the reactivity of diamondoids towards CH‐substitution.

### 2.4 Identification of Active Fungal Components

As brown‐rot fungi are known producers of ROS (reactive oxygen species), their presence in the media was analyzed. However, no significant amounts of ROS were found in the culture media (See Supporting Information, Scheme S.1). This is in agreement with the high acidity of growing fungal media, as low pH values contribute to binding of Fe^3+^ by oxalic acid and suppress its reduction to Fe^2+^ followed by HO radical generation [46].

Several experiments were conducted using active fungal components, specifically the supernatant and resting cells, collected on the 2^nd^, 4^th^, and 6^th^ culture days (Table 1). No product formation was detected in the supernatant samples. The mycelium was tested both with pure water and buffer solutions of varying pH values. The diols **7** and **8** were observed only in experiments in pure water with mycelium collected on days 4 and 6, and in the buffer at pH = 2 with resting cells collected on day 6. Although pure water was added to the resting cells, the pH of the resulting reaction mixture dropped to approximately 2, as the cells acidified the medium to conditions favorable for their activity. In contrast, the addition of a low‐pH buffer may act as an additional stress factor, while the lack of growth limits metabolic activity and cofactor synthesis required for cofactor‐dependent enzymes, which together lead to a decrease in catalytic activity. The loss of oxidative activity at higher pH values may be related to the pH dependence of enzymes’ activity that is typical for *W. cocos* cultures.

**TABLE 1:** Results of the biotransformation of adamantane‐1‐ol (**1**) with active fungal components collected on different culture days.

Fungal component		Culture day		
		2	4	6
Supernatant		—	—	—
Resting cells	H_2_O_dd_	—	Diol traces	Diols **7** (23%), **8** (traces)
	pH 2	—	—	Diols **7** (8%), **8** (traces)
	pH 4–8	—	—	—

## 3 Experimental Section

### 3.1 Organism

The filamentous fungus *Wolfiporia cocos* (CBS 279.55) used in this work was supplied by the Centraalbureau voor Schimmelcultures (CBS, Baarn, Netherlands). The stock culture was maintained on a solid medium containing 15 g L^−1^ malt extract (Fluka, Neu‐Ulm, Germany) and 15 g L^−1^ agar–agar (Roth, Karlsruhe, Germany).

### 3.2 Chemicals

Adamantane‐1‐ol (99%) and adamantane‐1,3‐diol (99%) were purchased from Sigma–Aldrich (St. Louis, Missouri, USA). 1,3‐dimethyladamantane (99%) was bought from TCI Deutschland GmbH (Eschborn, DE). 1‐bromoadamantane was purchased from BLD PHARMATECH GmbH (Kaiserslautern, DE). Diamantane‐1‐ol, diamantane‐4‐ol, 4‐bromodiamantane, and 4‐iododiamantane were synthesized according to [45, 47].

### 3.3 Submerged Cultures

The culture medium was prepared by dissolving malt extract (20 g) in 1 L of deionized water. For the preparation of the precultures, a 1 cm^2^ agar plug from the leading mycelial edge was transferred into 100 mL medium (in 250 mL Erlenmeyer flask) and then homogenized with a T 25 digital Ultra‐Turrax homogenizer (IKA, Staufen, Germany; 30 s, 10.000 r min^–1^). The precultures were grown on an incubation shaker (Orbitron, Infors HAT, Bottmingen, Switzerland; 150 r min^–1^, deflection 25 mm) under the exclusion of light at 24°C for 7 days. Subsequently, the precultures were homogenized, and 10% (v/v) of the homogenate was inoculated for submerged cultivation on 40 mL (in 100 mL Erlenmeyer flasks) and 200 mL (in 500 mL Erlenmeyer flasks) scales.

### 3.4 Screening of Fungal Activity

Substrate was added to submerged cultures of *W. cocos* grown in malt extract (2%) medium (40 mL) on the *X* (*X* = 1–7) culture day, to achieve final concentrations of 5, 10, 20, 30 mM. The reaction mixture was placed on an incubation shaker at 150 r min^−1^ under the exclusion of light at 24°C for 6 days. Samples were taken every 24 h and freeze‐dried (Vaco2, Zirbus Technology, Bad Grund, Germany). For analysis, methanol (2 mL) containing 1,3‐dimethyladamantane (2 mM) as internal standard was added, and the resulting mixture was shaken and centrifuged (4000 × g, 2 min, 4°C) to separate the phases. The organic phase was filtered over a cotton‐filled glass pipette and analyzed by GC–MS (Agilent Technologies 7890A GC, column Agilent VF‐WAXms (30 m × 0.25 mm, 0.25 µm) and an Agilent 5975C MSD Triple‐Axis mass spectrometer). Blank biotransformation reactions were performed with autoclaved cultures as control. Every experiment was performed in triplicate.

### 3.5 Biotransformation with Growing Cultures of *W.* *cocos*

Substrate (adamantane‐1‐ol (**1**), 1‐bromoadamantane (**2**), diamantane‐1‐ol (**3**), diamantane‐4‐ol (**4**), 4‐bromodiamantane (**5**), or 4‐iododiamantane (**6**)) (2 mmol, final concentration 10 mM) was added to submerged cultures of *W. cocos* grown in malt extract (2%) medium (200 mL) on the 4^th^ or 6^th^ culture day. The reaction mixture was placed on an incubation shaker at 150 r min^−1^ under the exclusion of light at 24°C for 6 days. For extraction, mycelium and supernatant were separated by centrifugation (4000 × g, 20 min, 4°C). The resulting supernatant was freeze‐dried (Vaco2, Zirbus Technology, Bad Grund, Germany). The dried extract was then redissolved in methanol. The resulting reaction mixture (1 g) was analyzed by GC–MS and NMR (Avance II 4 00 MHz WB (AV 400) and Avance III 600 MHz (AV 600)) spectroscopy. Products were purified by column chromatography on silica gel (adamantane derivatives: eluent (ethyl acetate/methanol) changed gradually, starting from 100% ethyl acetate, followed by 5/1, 1/1 (v/v), and finally 100% methanol; diamantane derivatives: eluent diethyl ether/ethyl acetate 9:1) to isolate the major components. The respective fractions were combined, concentrated in vacuum, and the ^1^H and ^13^C NMR spectra of the products were compared with those of standard samples.

Adamantane‐1,3‐diol (**7**) 235 mg (70%) and (*anti*)‐adamantane‐1,4‐diol (**8**) 61 mg (18%) were isolated in case of **1** as substrate.

Adamantane‐1‐ol (**1**) 30.4 mg (10%), adamantane‐1,3‐diol (**7**) 151 mg (70%), and (*anti*)‐adamantane‐1,4‐diol (**8**) 30.5 mg (18%) were isolated in case of **2** as substrate.

Diamantane‐4,9‐diol (**10**) 268 mg (61%) and diamantane‐1,9‐diol (**11**) 123 mg (28%) were isolated in case of **4** as substrate.

#### 3.5.1 Adamantane‐1,3‐diol

^1^H NMR (400 MHz, DMSO‐d_6_, ppm): *δ* = 4.50 (s, 2H), 2.12 (s, 2H), 1.49–1.40 (m, 10H), 1.36 (s, 2H). ^13^C NMR (100 MHz, DMSO‐d_6_, ppm): *δ* = 69.00 (2C), 53.69 (CH_2_), 44.50 (4CH_2_), 35.17 (CH_2_), 31.12 (2CH). MS (m/z): 168 (14%), 112 (9%), 111 (100%), 110 (5%), 109 (5%), 108 (5%), 95 (15%), 94 (5%), 93 (4%), and 55 (5%).

#### 3.5.2 (*Anti*)‐Adamantane‐1,4‐diol

^1^H NMR (400 MHz, MeOD, ppm): *δ* = 3.73 (m, 1H), 2.00–1.93 (m, 3H), 1.87 (s, 2H), 1.68–1.57 (m, 6H), 1.26 (d, *J* = 12 Hz, 2H). ^13^C NMR (100 MHz, MeOD, ppm): *δ* = 72.84 (CH–OH), 66.63 (C), 44.75 (CH_2_), 42.88 (2CH_2_), 36.00 (2CH), 30.01 (CH), 29.39 (2CH_2_). MS (m/z): 168 (30%), 150 (8%), 110 (100%), and 95 (99%).

#### 3.5.3 Diamantane‐4,9‐diol

^1^H NMR (400 MHz, MeOD/CDCl_3_ 1/1, ppm): *δ* = 1.83 (s, 6H), 1.65 (s, 12H). ^13^C NMR (100 MHz, MeOD/CDCl_3_ 1/1, ppm): *δ* = 66.46 (2C), 44.00 (6CH_2_), 38.69 (6CH). MS (m/z): 221 (14%), 220 (100%), 110 (12%), 109 (13%), 108 (14%), 107 (33%), 95 (16%), 94 (8%), 93 (7%), 91 (9%), and 77 (9%).

#### 3.5.4 Diamantane‐1,9‐diol

M.p. = 203–204°C. ^1^H NMR (400 MHz, CDCl_3_, ppm): *δ* = 2.11–2.00 (m, 4H), 1.70–1.66 (m, 1H), 1.65–1.61 (m, 4H), 1.60–1.57 (m, 4H), 1.55–1.51 (m, 4H), 1.50–1.39 (m, 3H). ^13^C NMR (100 MHz, CDCl_3_, ppm): *δ* = 71.10 (C–OH), 69.19 (C–OH), 52.49 (CH_2_), 43.88 (2CH_2_), 41.01 (2CH), 39.67 (2CH), 35.86 (CH_2_), 34.38 (CH), 30.71 (2CH_2_), 23.90 (CH). MS (m/z): 220 (18%), 202 (50%), 147 (20%), 124 (29%), 123 (100%), 112 (18%), 111 (21%), 110 (39%), 107 (15%), 95 (12%), 93 (15%), 92 (16%), 91 (18%), 77 (14%). HRMS: found 220.1466 (calculated for C_14_H_20_O_2_ 220.1463).

### 3.6 Biotransformation Using Resting Cells of *W. cocos*

The mycelium was collected from the submerged cultures of *W. cocos* (200 mL) after 4 or 6 culture days. Sterilized McIlvaine buffer (0.1 M, pH = 2.1) was added (5 mL) to the separated mycelium prior to the supplementation with the substrate (5 mM). Additionally, experiments were performed using the separated mycelium without buffer addition to evaluate the influence of the buffer system on the biotransformation process. The reaction mixtures were placed on an incubation shaker at 150 r min^−1^ (deflection 25 mm) under exclusion of light at 24°C for 24 h. After 24 h, 0.5 g of NaCl was added to the reaction mixture and shaken. For extraction, 2 mL Et_2_O containing 1,3‐dimethyladamantane (2 mM) as internal standard was added. The resulting mixture was shaken and centrifuged (4000 × g, 2 min, 4°C) to separate the phases. The extraction process was repeated three times. The combined organic phases were dried over Na_2_SO_4_ and analyzed using GC–MS. Every biotransformation was repeated three times to verify the reproducibility of the experiments.

### 3.7 Biotransformation Using Supernatant of *W. cocos*

The supernatant was collected from the submerged cultures of *W. cocos* (200 mL) after 4 or 6 culture days. Supernatant samples were either used directly or fractionated by centrifugal ultrafiltration through 10 kDa MWCO filters (Amicon Ultra centrifugal filters) to obtain a permeate (<10 kDa) and a retentate (>10 kDa). The substrate (5 mM) was added to each tested fraction. The reaction mixtures were placed on an incubation shaker at 150 r min^−1^ (deflection 25 mm) under exclusion of light at 24°C for 24 h. After 24 h, the reaction mixture was processed as described in Section 3.6. and analyzed using GC–MS. Every biotransformation was repeated three times to verify the reproducibility of the experiments.

### 3.8 Measurement of Extracellular Superoxide Anion Radical Content

The level of superoxide anion radicals was assessed spectrophotometrically by measuring of superoxide anion‐dependent formation of formazone from nitrotetrazolium blue (NBT) in alkaline medium [48]. The reaction mixture consisted of 3 mL of distilled water, 50 µL of 1 M NaOH, and 100 µL of 5 mM NBT solution, to which 100 µL of the sample was added. After incubation at room temperature for 10 min, the absorbance was measured at 560 nm. The superoxide anion content of the mycelial samples was expressed as a function of absorbance reading at 560 nm.

## 4 Conclusion

The oxidative behavior of *W. cocos* is strongly influenced by culture age and reaction conditions. Screening experiments revealed distinct substrate preferences depending on the developmental stage of the culture. 4‐D‐old submerged cultures exhibited the highest activity toward adamantane‐1‐ol, whereas 6‐d‐old cultures showed superior performance in the oxidation of 1‐bromoadamantane as highly acidic media promote hydrolysis. In the case of diamantane derivatives, diamantane‐1‐ol exhibited approximately 20% conversion, yielding a complex mixture of diols and triols. In contrast, the apical isomer, diamantane‐4‐ol, underwent almost complete conversion, producing predominantly diamantane‐4,9‐diol and unexpectedly diamantane‐1,9‐diol. This indicates a selective shift in oxidation patterns, likely reflecting the enzymatic specificity of the fungal system.

The results clearly showed that oxidative activity of *W. cocos* resided primarily in the mycelial biomass rather than in the extracellular medium. Resting cells from 4‐ and 6‐d‐old cultures retained activity in pure water medium, while additional activity was observed in 6‐d‐old cultures in buffer at pH 2. However, this oxidative capacity was lost under higher pH values. These enzymes could represent compelling targets for directed evolution or genetic engineering to optimize their activity and selectivity towards various adamantane derivatives, thereby enabling the tailored synthesis of specific diamondoid diols with high efficiency.

Overall, the findings confirm that *W. cocos* possesses an efficient and selective oxidative system capable of catalyzing the transformation of rigid polycyclic hydrocarbon derivatives. The study highlights the potential of this basidiomycete as a sustainable biocatalyst for the regioselective oxidation of structurally complex substrates under mild and environmentally benign conditions. We also found the superiority of diamantane derivatives for mechanistic studies where the difference between fungal and traditional CH‐activation chemistries becomes clear even at synthetic level.

## Supporting Information

Additional supporting information can be found online in the Supporting Information section. **Supporting Fig. S1.1:** Time course of 1,3‐adamantanediol production in biotransformation reactions with whole‐cell growing cultures of *W. cocos*, using varying concentrations of 1‐adamantanol (5 mM, 10 mM, 20 mM, and 30 mM). Substrate 1 was added to the cultures on the 1^st^ (**A**) and 2^nd^ (**B**) day. Error bars represent standard deviations based on triplicate experiments. **Supporting Fig. S1.2:** Time course of 1,3‐adamantanediol (A) and 1‐adamantanol (B) production in biotransformation reactions with 4‐day‐old cultures of *W. cocos* with varying concentrations of **2** (5 mM, 10 mM). Error bars represent standard deviations based on triplicate experiments. **Supporting Fig. S1.3:** Superoxide anion production in biotransformation reactions with whole‐cell growing cultures of *W. cocos* measured in samples collected on the 1^st^‐6^th^ days. Substrate **1** concentration was 5 mM (**A**), 10 mM (**B**), 20 mM (**C**), 30 mM (**D**). **Supporting Fig. S1.4:** Changes in pH during the growth of *W. cocos*. (**A**) Control without substrate addition. (B–H) pH changes after substrate addition (5 mM) on day 1 (**B**), day 2 (**C**), day 3 (**D**), day 4 (**E**), day 5 (**F**), day 6 (**G**), and day 7 (**H**) of fungal growth. Samples were collected daily from day 1 to day 6. **Supporting Fig. S2.1:** ^1^H NMR of 1,3‐adamantanediol (**7**). **Supporting** **Fig.** **S2.2:** ^13^C NMR of 1,3‐adamantanediol (**7**). **Supporting Fig. S2.3:** ^1^H NMR of 1,4‐(*anti*)‐adamantanediol (**8**). **Supporting Fig. S2.4:** ^13^C NMR of 1,4‐(anti)‐adamantanediol (**8**). **Supporting Fig. S2.5:** ^1^H NMR of 4,9‐diamantanediol (**10**). **Supporting Fig. S2.6:** ^13^C NMR of 4,9‐diamantanediol (**10**). **Supporting Fig. S2.7:** ^1^H NMR of 1,9‐diamantanediol (**11**). **Supporting Fig. S2.8:** ^13^C NMR of 1,9‐diamantanediol (**11**).

## Funding

This study was supported by Alexander von Humboldt‐Stiftung.

## Conflicts of Interest

The authors declare no conflicts of interest.

## Supporting information

Supplementary Material

## Data Availability

The data that support the findings of this study are available from the corresponding author upon reasonable request.
