# Supplementary material for: Biocatalytic Synthesis of Diamondoid Diols by the Brown‐Rot Fungus Wolfiporia cocos
Source: Chembiochem. 2026 Feb 1;27(3):e202500930. doi: 10.1002/cbic.202500930 (PMC12862244; doi:10.1002/cbic.202500930)
Supplement: Supplementary file 1 — Supplementary Material [file CBIC-27-e202500930-s001.pdf]

Supplementary information

Biocatalytic Synthesis of Diamondoid Diols by the Brown-Rot Fungus *Wolfiporia cocos*

V.V. Nikitenkova [a], *A.M. Sydorenko* [b], *H. Zorn* [a,c], *T.S. Zhuk* [a,b*]

[a]Institute of Food Chemistry and Food Biotechnology, Justus Liebig University Giessen

Heinrich-Buff-Ring, 17, 35392 Giessen, Germany

[b]Faculty of Chemical Technology, Igor Sikorsky Kyiv Polytechnic Institute

Beresteiskyi Ave, 37, Kyiv, Ukraine, 03056

[c]Fraunhofer Institute of Molecular Biology and Applied Ecology

Ohlebergsweg, 12, 35392, Giessen, Germany

*Corresponding author: Faculty of Chemical Technology, Igor Sikorsky Kyiv Polytechnic Institute, Beresteiskyi Ave., 37, 03056 Kyiv, Ukraine; t.zhuk@xtf.kpi.ua (Tatyana Zhuk)

Table of content

1. **Experimental details** S3

1.1. Details of biotransformations with 1-day-old and 2-da-old cultures of *W. cocos* S3

1.2. Details of biotransformations with 4-day-old cultures of *W. cocos* S3

1.3. Details of the measurement of extracellular superoxide anion radical content S4

1.4. Details of the pH values S5

1.5. Details of biotransformation with growing culture of *W. cocos* in the presence of organic solvents S6

2. **NMR spectra of isolated products** S7

1. Experimental details

1.1. Details of biotransformations with 1-day-old and 2-da-old cultures of *W. cocos*

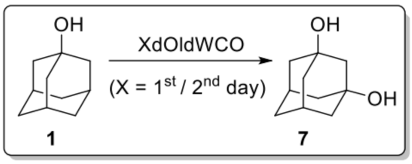

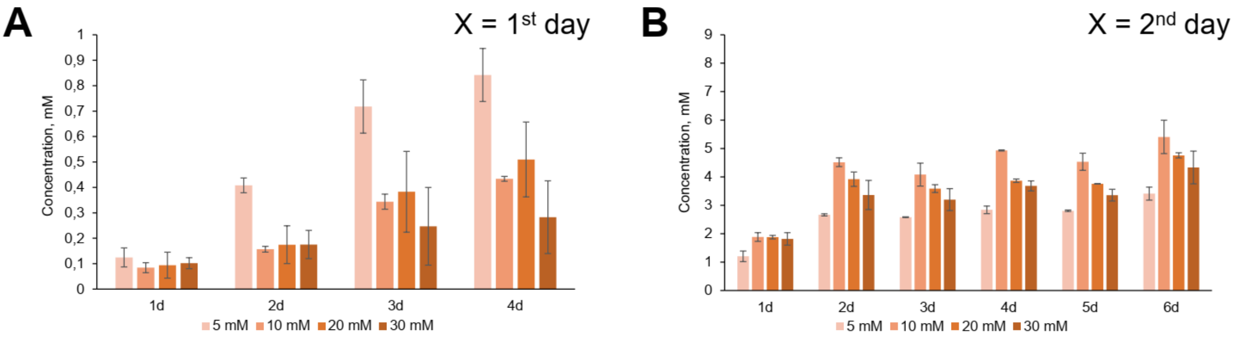

Figure S1.1. Time course of 1,3-adamantanediol production in biotransformation reactions with whole-cell growing cultures of *W. cocos*, using varying concentrations of 1-adamantanol (5 mM, 10 mM, 20 mM, and 30 mM). Substrate 1 was added to the cultures on the 1st (A) and 2nd (B) day. Error bars represent standard deviations based on triplicate experiments..

1.2. Details of biotransformations with 4-day-old cultures of *W. cocos*

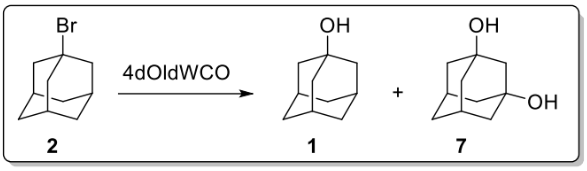

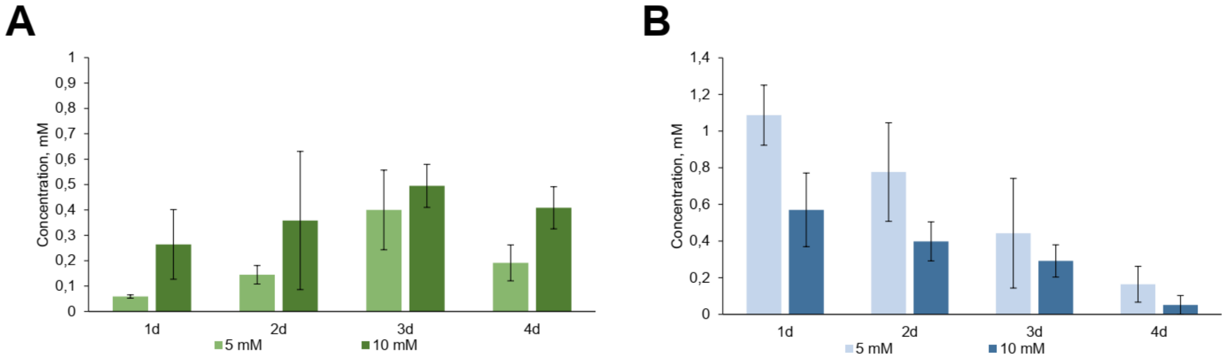

Figure S1.2. Time course of 1,3-adamantanediol (**A**) and 1-adamantanol (**B**) production in biotransformation reactions with 4-day-old cultures of *W. cocos* with varying concentrations of **2** (5 mM, 10 mM). Error bars represent standard deviations based on triplicate experiments.

1.3. Details of the measurement of extracellular superoxide anion radical content

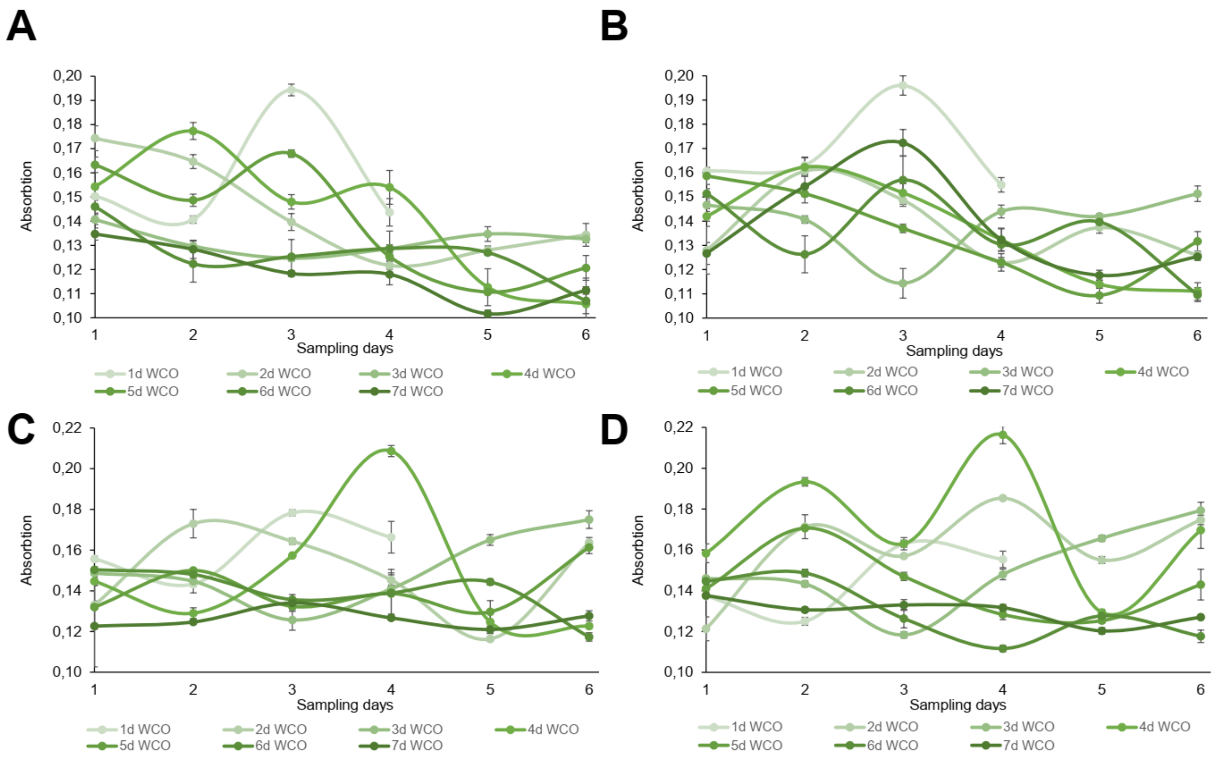

Figure S1.3. Superoxide anion production in biotransformation reactions with whole-cell growing cultures of *W. cocos* measured in samples collected on the 1st-6th days. Substrate **1** concentration was 5 mM (**A**), 10 mM (**B**), 20 mM (**C**), 30 mM (**D**).

1.4. Details of the pH values

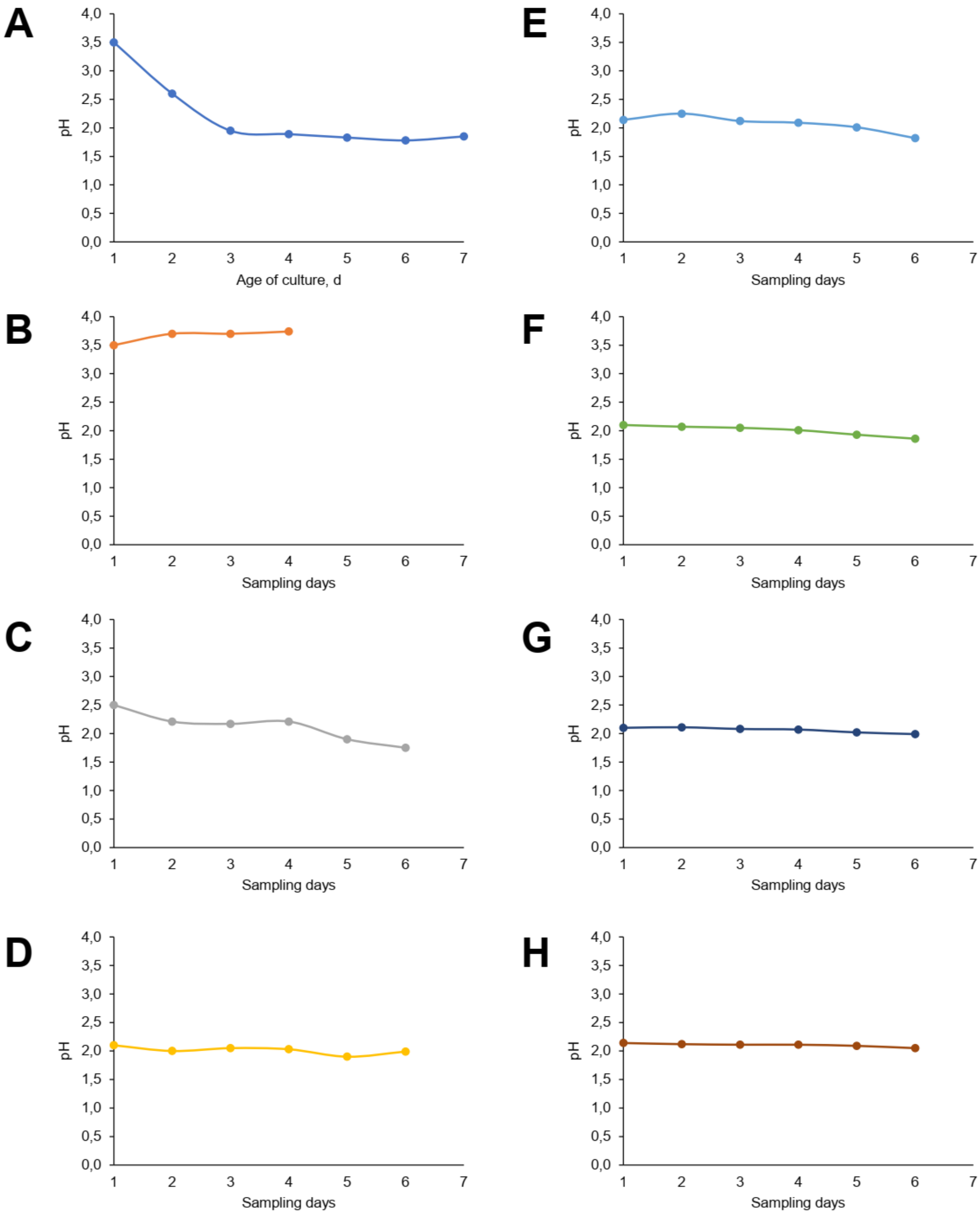

Figure S1.4. Changes in pH during the growth of *W. cocos*. (**A**) Control without substrate addition. (B–H) pH changes after substrate addition (5 mM) on day 1 (**B**), day 2 (**C**), day 3 (**D**), day 4 (**E**), day 5 (**F**), day 6 (**G**), and day 7 (**H**) of fungal growth. Samples were collected daily from day 1 to day 6.

1.5. Details of biotransformation with growing culture of *W. cocos* in the presence of organic solvents

Substrate (adamantane-1-ol (**1**) or 1-bromoadamantane (**2**)) was added to submerged cultures of *W. cocos* (40 mL) on the 4th and 6th culture day, and the biotransformation proceeded for 4 days. Substrate was added as 1 mL of 400 mM solution (2.5% of cosolvent) or 1 mL of 100 mM solution (10% of cosolvent) to a final concentration of 10 mM. Ethanol, 2-propanol and dimethyl sulfoxide were tested as cosolvents. Sample were taken every 24 h and freeze-dried. For analysis, methanol (2 mL) containing 1,3-dimethyladamantane (2 mM) as internal standard was added, and the resulting mixture was shaken and centrifuged (4000 x g, 2 min, 4 °C) to separate the phases. The organic phase was filtered over a cotton-filled glass pipette and analyzed by GC-MS. Every experiment was repeated three times to verify the reproducibility of the experiments.

2. NMR spectra of isolated products

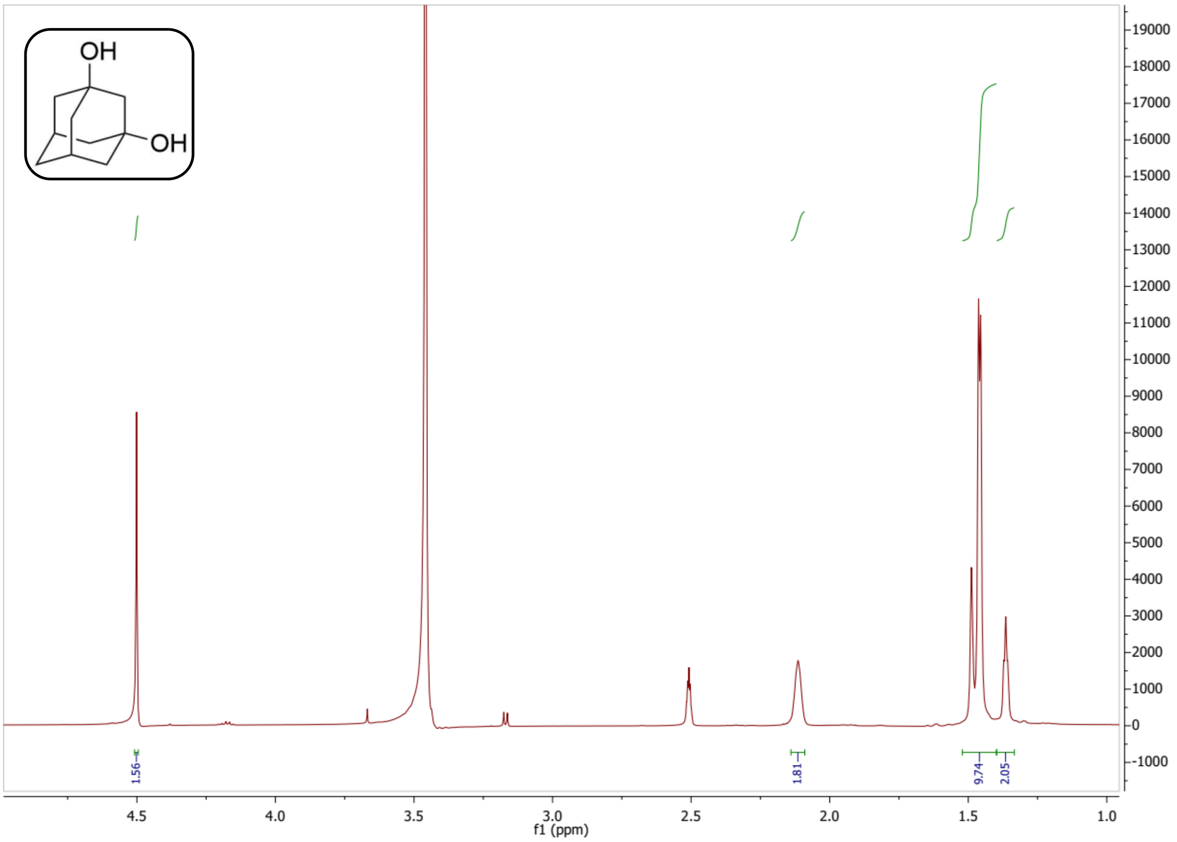

Figure S2.1. ^{1}H NMR of 1,3-adamantanediol (**7**).

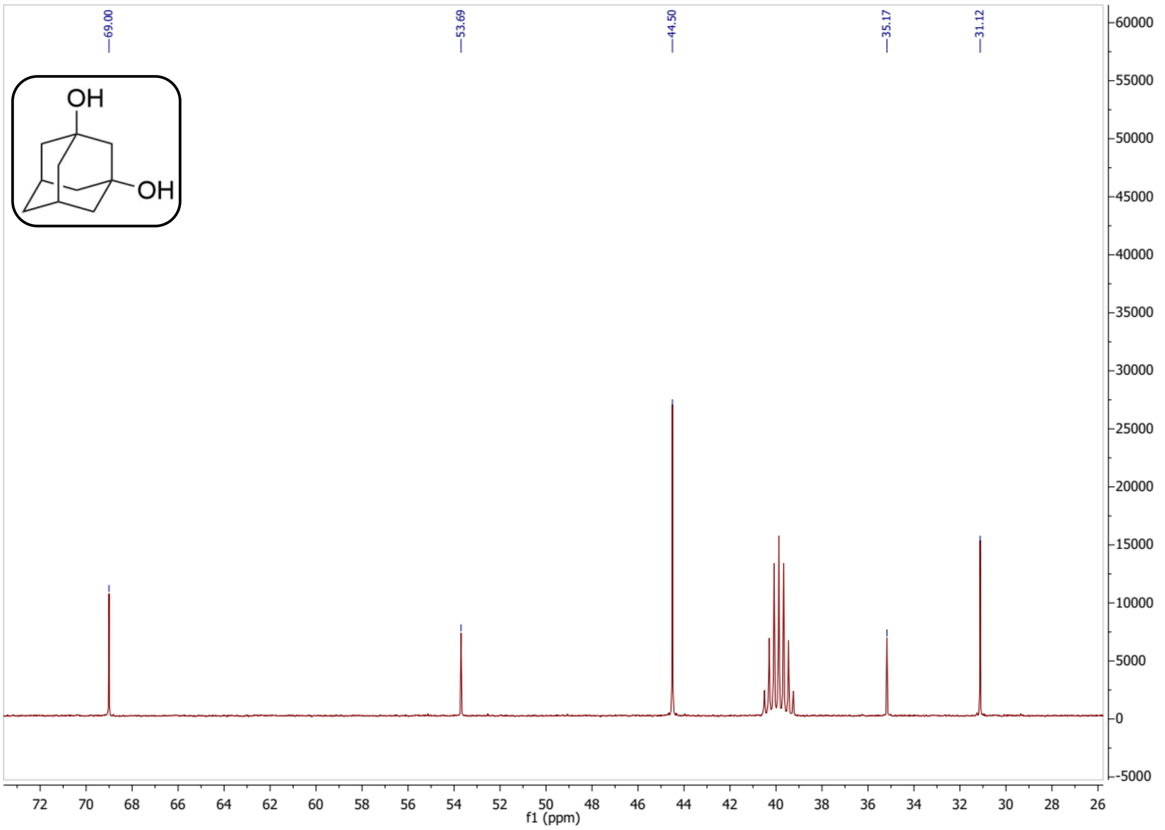

Figure S2.2. ^{13}C NMR of 1,3-adamantanediol (**7**).

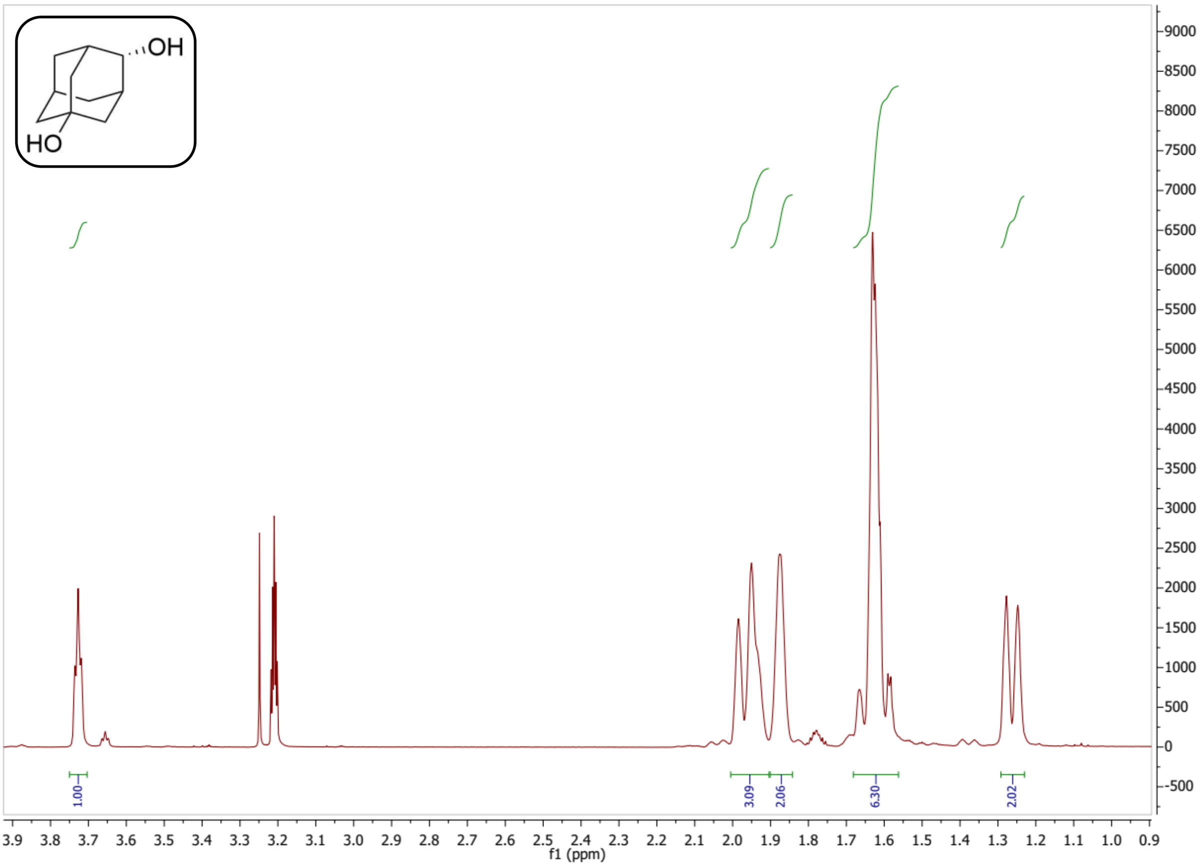

Figure S2.3. ^{1}H NMR of 1,4-(*anti*)-adamantanediol (**8**).

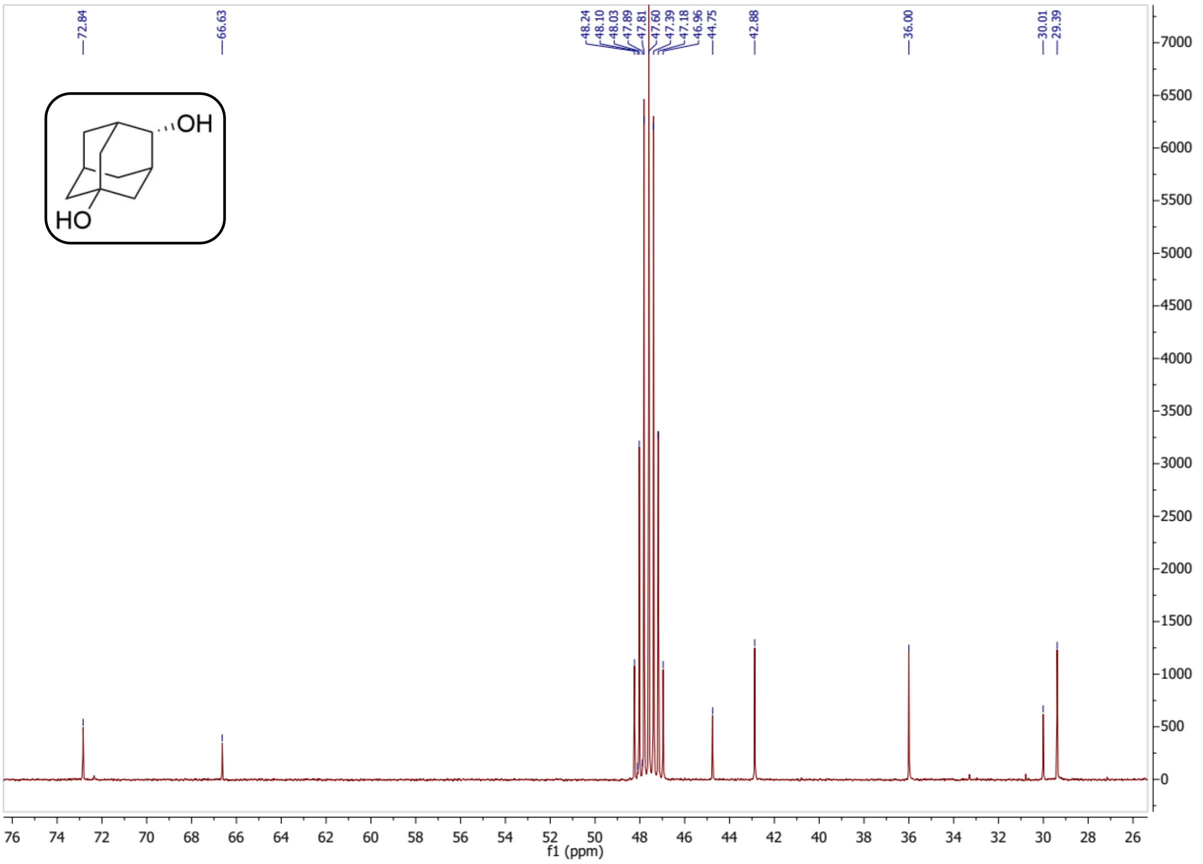

Figure S2.4. ^{13}C NMR of 1,4-(*anti*)-adamantanediol (**8**).

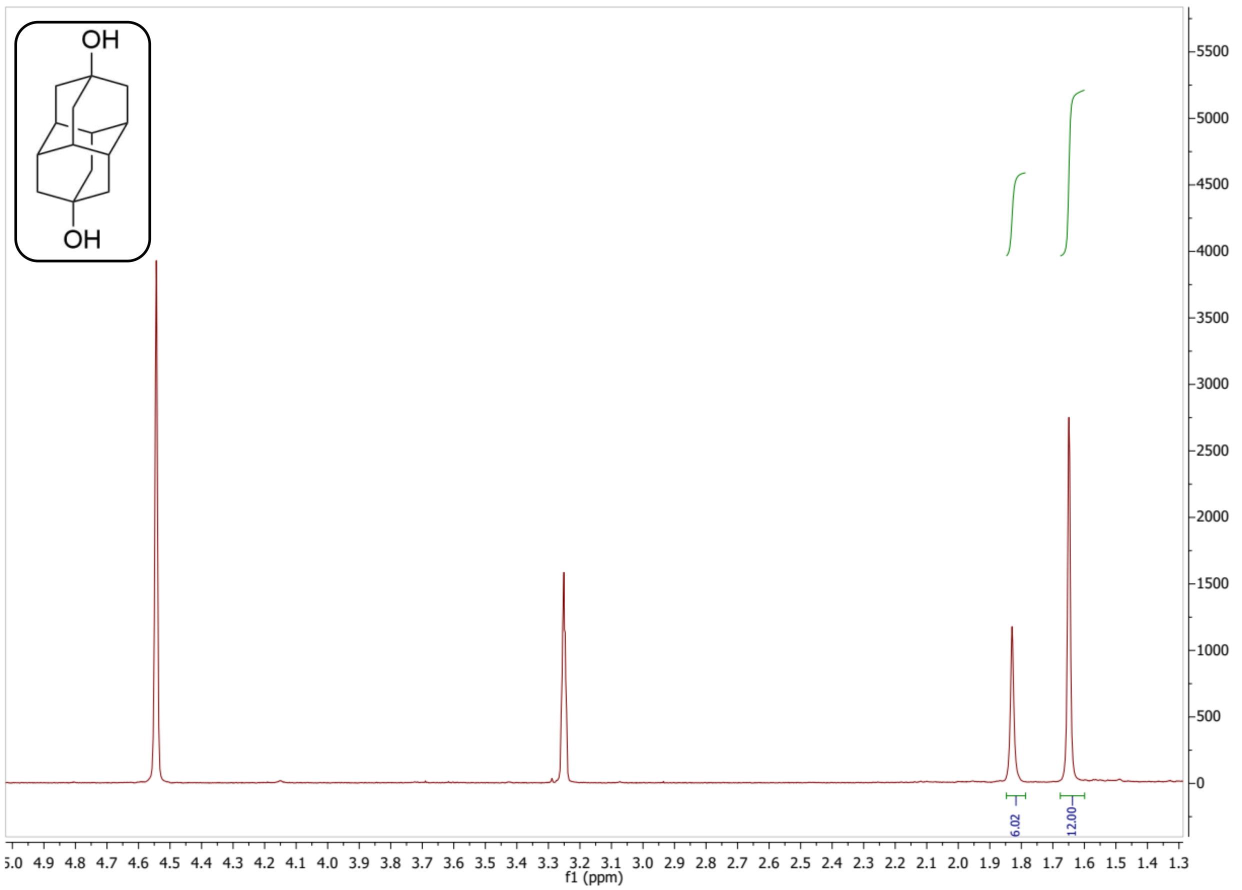

Figure S2.5. ^{1}H NMR of 4,9-diamantanediol (**10**).

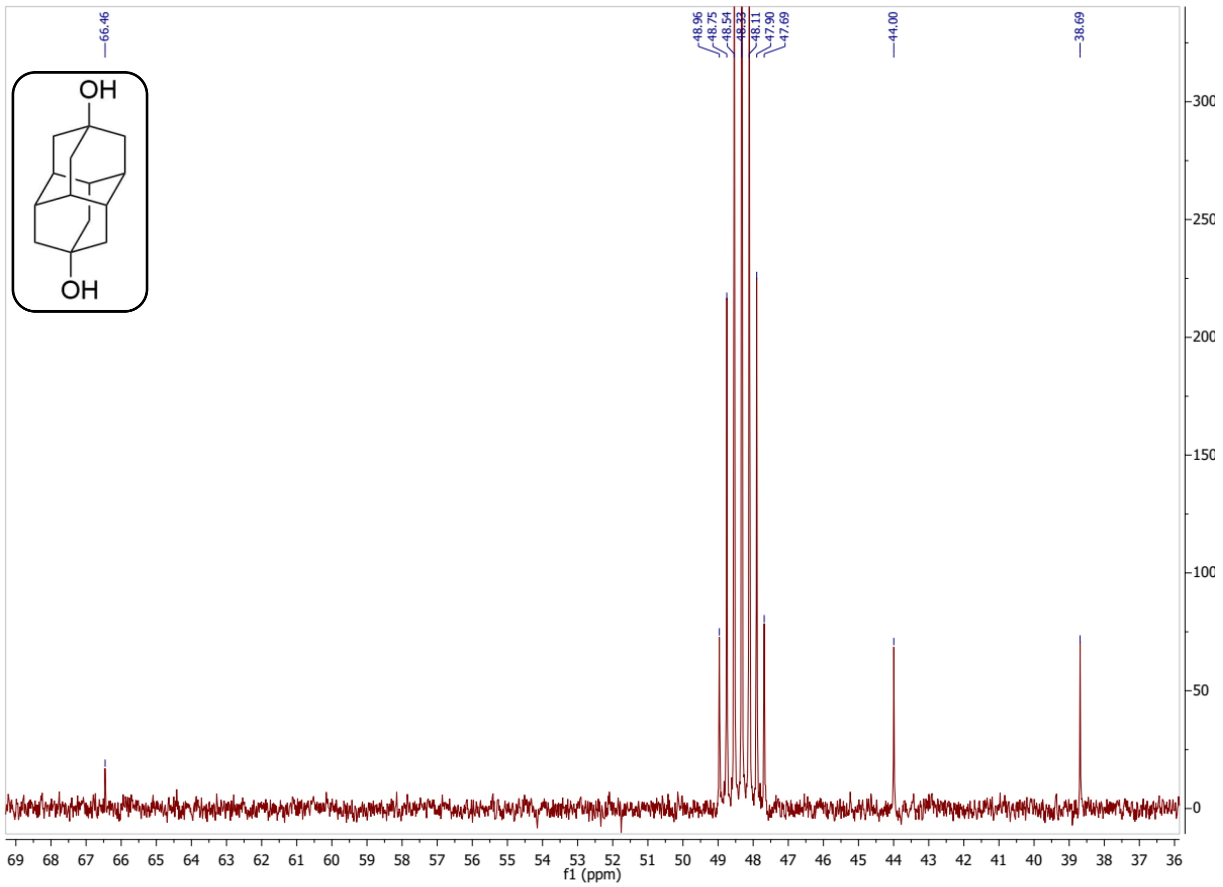

Figure S2.6. ^{13}C NMR of 4,9-diamantanediol (**10**).

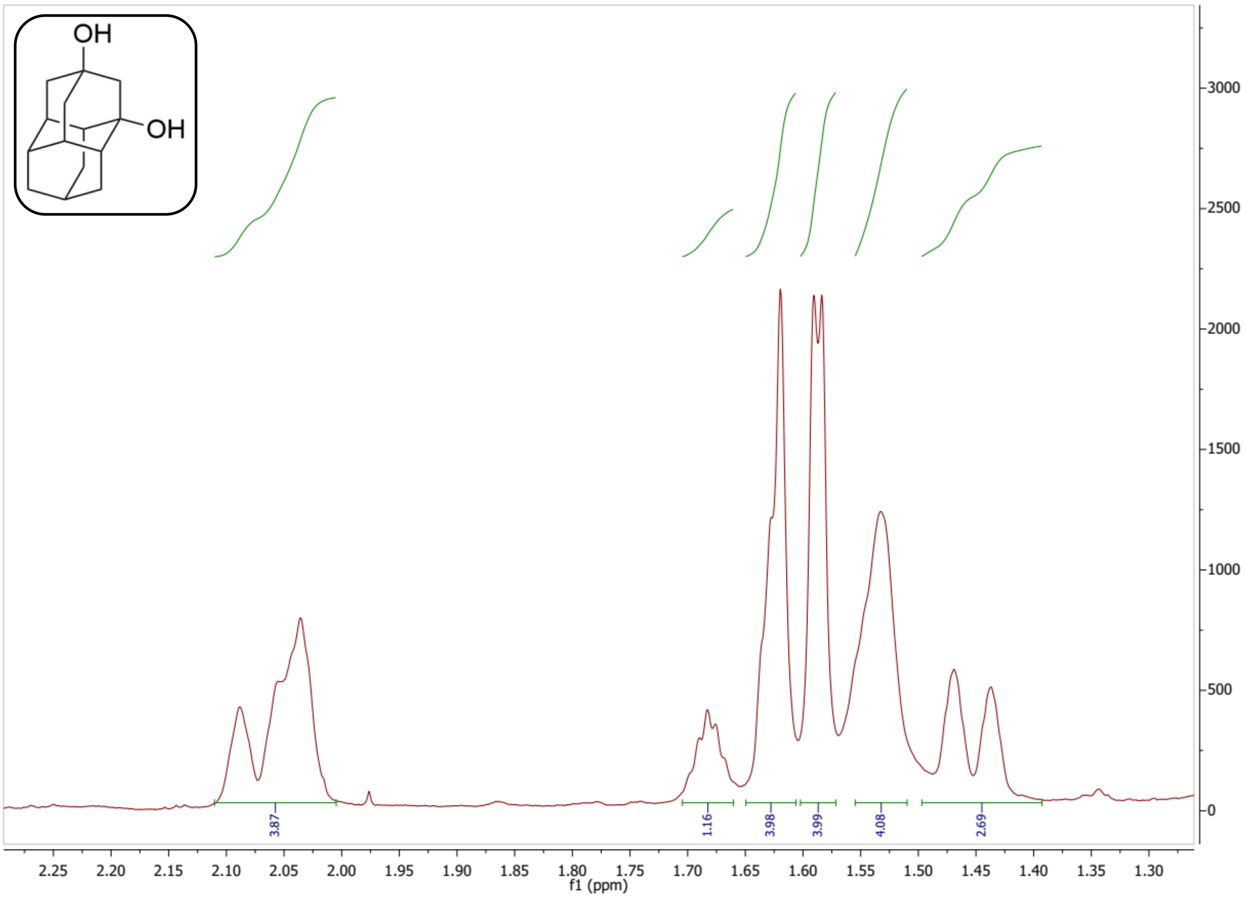

Figure S2.7. ^{1}H NMR of 1,9-diamantanediol (**11**).

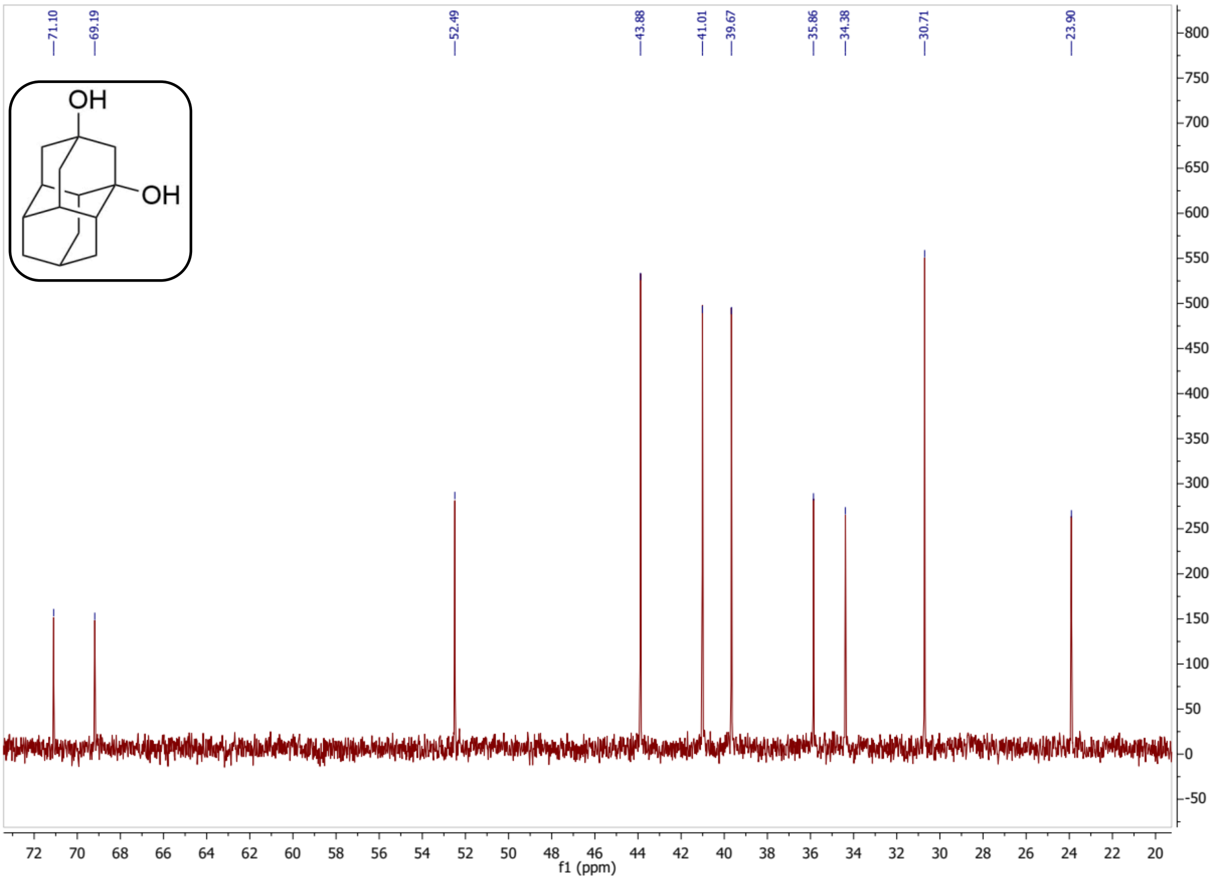

Figure S2.8. ^{13}C NMR of 1,9-diamantanediol (**11**).
